# A New Race in Egypt

**Published:** 1895-09-14

**Authors:** 


### THE STUDY OF MAN.

### IX.—A NEW RACE IN EGYPT.

It is not often that it falls to the lot of an explorer, not only to discover in a single season's work abundant remains of a new and distinct type of civilisation, but to find reason to attribute it to a new race of men, originating in an unsuspected quarter, and spreading over an area which had already, apparently, been adequately examined; and further to be able at once to give an intelligible account of the new facts, and to fix with some precision the relations of the newly-found race to the peoples who preceded and succeeded it, and at least the general chronological limits of its appearance.

Such, however, has been the lot of Professor Flinders Petrie and his colleagues, whose excavations in Upper Egypt during the past winter have not only filled, at all events in outline, one of the worst gaps in Egyptian history, but have added a series of observations of first-rate importance to our knowledge of the ethnology of North-East Africa. The results of the expedition have been exhibited during the month of July in the Egyptian Department of University College in Gower Street, and form one of the principal subjects of discussion at the meeting of the British Association now proceeding at Ipswich.

The sites which have yielded these important collections lie on the edge of the Libyan desert, above the western bank of the Nile, some thirty miles below Luxor and the ruins of Thebes. Two large cemeteries of the new race have been thoroughly explored, and have yielded some 2,000 tombs; and, besides this, the remains of two considerable settlements of the same people have been identified, full of objects of exactly similar character to those found in their tombs.

The relative age of this civilisation is fixed by the fact that some of the tombs break through into recognisable Egyptian mastaba-sepulchres of the Fourth Dynasty, while others are themselves overlaid and disturbed by burials of the Twelfth Dynasty; some of the latter also occur in and over the ruins of the settlement itself, which must, therefore, have been then already quite deserted.

Now this period of Egyptian history, extending from the Fourth and Fifth to the Twelfth Dynasty, has been hitherto an almost complete blank. Though the names of the kings of the intermediate dynasties are traditionally known, almost no monuments remain which can be attributed to them, and all that is preserved of their history is a hint that their dominion was limited to Middle Egypt, and the fact that the Sixth Dynasty seems to perish in some national disaster, and that the Eleventh appears to represent the beginning of a national recovery from a period of prostration. Professor Petrie claims that in the discovery of the new race we have a clue to the mystery. He represents a people (who were, to judge from their skeletons and from the portraits of themselves which adorn their furniture, of North African origin and affinities) sweeping eastwards into Egypt, overwhelming the Old Kingdom and extending perhaps even into Syria.

From the fact that they borrowed nothing either of culture or of the products of industry from the Egyptians, it seems probable that the invaders absolutely exterminated the natives from the area which they occupied; for had they preserved any of their captives, even as slaves, it would be almost impossible that some trace of Egyptian handiwork should not have been recognisable in the fashion of their pottery and ornaments. As it is, even the use of the potter's wheel remained quite unknown, and all their vessels, wonderfully symmetrical as they are, are modelled wholly by the unassisted fingers, like the primitive pottery of Syria and of Cyprus, where their characteristic red-polished surface is also very closely paralleled.

The survivors, on the other hand, of the native Egyptians seem to have borrowed not a little from the new comers, though more in the way of single manufactured objects, which are occasionally found almost all over Middle Egypt, than by adopting from them any special art or skill.

The manufacture, however, of stone vases, which has been long noted as apparently beginning under the Twelfth Dynasty, may very probably be considered as a loan from the new race, who excelled in this art.

Their proficiency in stone work, indeed, is one of their most characteristic features; even the long series of vases cut from marble and slate, and even from harder stones, like diorite and basalt, is far surpassed by the delicacy and beauty of their flint working. Daggers and spearheads, sometimes six or eight inches long, and as thin as a paper-knife, and bangles large enough to be worn, yet less than half an inch in width, are cuts from the compact flints of the desert and the gravels, and finished by flaking or sand-polishing with the greatest precision. Most peculiar among them are the forked spear heads, to be thrown among the legs of deer to bring them to the ground.

But though they excelled in the manipulation of stone and continued to use it commonly until their history closes, they were by no means unacquainted with the use of metal. Copper was used, almost without alloy, for adzes, harpoons, and needles. The latter are of some delicacy and are accompanied still with thread and fragments of textiles. The forms of the weapons, however, are quite different from the Egyptian types; and one of them, a fine bayonet-bladed dagger, resembles far more nearly a type which is characteristic of the prehistoric Mykenæan civilisation of Greece and the Ægean Islands, which is already known to owe not a little to Egyptian sources of the Twelfth Dynasty or earlier.

It is impossible as yet to anticipate the more detailed study which will make the whole life and history of this "new race" intelligible, or to do justice to the variety and completeness of the collections which have been already made. It is enough to point out their extreme intrinsic interest and their great importance as a contribution to the early history of Egypt and to the ethnology of that part of Africa.

